# Quackery and the Legislature of Ohio

**Published:** 1849-10

**Authors:** 


### QUACKERY AND THE LEGISLATURE OF OHIO.

Under this head, the Ohio Medical Journal noticed some time ago the triumph of the Medical College of Ohio over its neighbors, the Thompsonians, Eclectics, and Homœopathists. The latter combined in an effort to obtain access to the Cincinnati Hospital, at present granted by the legislature exclusively to the Ohio Medical College. The privilege asked for was voted by the House, but the Senate wisely rejected the bill. All friends of medical science will rejoice at this result. Quackery in that vote of the Senate of Ohio received a merited rebuke.

